# Comparative transcriptome analysis of *Sogatella furcifera* (Horváth) exposed to different insecticides

**DOI:** 10.1038/s41598-018-27062-4

**Published:** 2018-06-08

**Authors:** Cao Zhou, Hong Yang, Zhao Wang, Gui-yun Long, Dao-chao Jin

**Affiliations:** 1 Institute of Entomology, Guizhou University; Provincial Key Laboratory for Agricultural Pest Management of Mountainous Regions, Guiyang, 550025 P. R. China; 2 College of Tobacco Science of Guizhou University, Guiyang, 550025 P. R. China; 3 College of Environment and Life Sciences, Kaili University, Kaili, 556011 P. R. China

## Abstract

White-backed planthopper, *Sogatella furcifera* (Horváth) (Hemiptera: Delphacidae), one of the main agricultural insect pests in China, is resistant to a wide variety of insecticides. We used transcriptome analysis to compare the expression patterns of resistance- and stress-response genes in *S*. *furcifera* subjected to imidacloprid, deltamethrin, and triazophos stress, to determine the molecular mechanisms of resistance to these insecticides. A comparative analysis of gene expression under imidacloprid, deltamethrin, and triazophos stress revealed 1,123, 841, and 316 upregulated unigenes, respectively, compared to the control. These upregulated genes included seven P450s (two CYP2 clade, three CYP3 clade, and two CYP4 clade), one GST, one ABC transporter (ABCF), and seven Hsps (one 90 and six Hsp70s) under imidacloprid stress; one P450 (CYP3 clade), two ABC transporters (one ABCF and one ABCD), and one Hsp (Hsp90) under deltamethrin stress; one P450 (CYP3 clade) and one ABC transporter (ABCF) under triazophos stress. In addition, 80 genes were commonly upregulated in response to the three insecticide treatments, including laminin, larval cuticle protein, and fasciclin, which are associated with epidermal formation. These results provide a valuable resource for the molecular characterisation of insecticide action in *S*. *furcifera*, especially the molecular characteristics of insecticide cross resistance.

## Introduction

The white-backed planthopper (WBPH), *Sogatella furcifera* (Horváth) (Hemiptera: Delphacidae) is a major pest in rice paddies throughout Asia. *S*. *furcifera* causes damage to rice by sucking phloem sap and transmitting Southern rice black-streaked dwarf virus^1^. The main method used to control the planthopper is the application of chemicals. However, the long-term application of chemicals will inevitably lead to resistance, planthopper resurgence, and environmental pollution^2^. Although the ecological mechanism of insecticide resistance has been studied extensively, the physiological mechanism of *S*. *furcifera* resistance remains unclear. We aimed to elucidate the mechanism of insecticide resistance by investigating changes in gene expression.

The broad-spectrum insecticides imidacloprid and triazophos have been widely used to control rice planthopper. However, the application of triazophos can significantly stimulate *Nilaparvata lugens* and *S*. *furcifera* fecundity, leading to their resurgence^3,4^. Rice planthopper resistance has been reported following the long-term and widespread spraying of imidacloprid in the field^5^, and it was suggested that its use to control *N*. *lugens* in China was ceased. Deltamethrin is highly effective in controlling rice stem borers adult, whereas rice planthoppers show high levels of natural resistance. Therefore, if the same concentration of deltamethrin used in the control of rice stem borer is used, rice planthoppers are likely to be only exposed to sublethal concentrations^6,7^. Although deltamethrin has been banned in China to control rice pests, it can still be used to control rice borers in many countries in Southeast Asia.

Enhanced biodegradation of insecticides is mainly due to the overexpression of genes encoding detoxifying enzymes, including glutathione S-transferases (GSTs), cytochrome P450 monooxygenases (P450s), and carboxylesterases (COEs)^8^. GSTs and P450s are large families of multifunctional enzymes involved in the detoxification of a wide range of xenobiotics, including insecticides^9,10^. Previously, eight P450 genes were found to be significantly overexpressed in susceptible strains of *N*. *lugens* following imidacloprid treatment (LD_50_)^11^. Compared with the susceptible strain, seven GST genes were overexpressed in a resistant strain, and silencing GST14 increased susceptibility to thiamethoxam in *Bemisia tabaci* Q^12^. Carboxylesterases play important roles in resistance via sequestration and hydrolysis^8^. Studies have shown that ATP-binding cassette (ABC) transporters and heat shock proteins (Hsps) are also associated with insecticide resistance. ABC transporters are membrane-bound proteins involved in the movement of various substrates, including drugs and insecticides^13^. Overexpression of ABC transporters has been associated with the development of insecticide resistance in many insect species. Quantitative PCR revealed that eight ABC transporters from the ABCB/C/D/G subfamilies were overexpressed in related strains of *Laodelphax striatellus* resistant to chlorpyrifos, deltamethrin, and imidacloprid, compared to in the susceptible strain^14^. Hsps are induced in response to stress induced by environmental factors and may be involved in adverse reactions, including insecticide resistance^15^.

Although the mechanisms of insecticide resistance have been reported previously, further research, especially on the common resistance mechanisms of pests to different insecticides, is needed before they can be fully elucidated. We used the Illumina HiSeq2000 (Illumina, San Diego, CA, USA) to produce a *de novo* transcriptome, and the BGIseq-500 (BGI, Wuhan, China; http://www.seq500.com/en/) platform to quantify expression levels of *S*. *furcifera* genes as a resource to provide insights into insecticide-related changes in gene expression following treatment with imidacloprid, triazophos, and deltamethrin. Furthermore, we aimed to identify a wide range of genes, including those encoding detoxification enzymes induced by insecticides used for the control of *S*. *furcifera*, and at the same time lay a foundation for understanding the resistance mechanisms of pests to different types of insecticides.

## Results

### Bioassay

The efficacy of three insecticides was tested on fifth-instar first-day nymphs using the rice stem dipping method. As shown in Table 1, the LC_10_ of imidacloprid, triazophos, and deltamethrin was 0.506, 11.581, and 8.276 mg/L, respectively.

**Table 1:** Toxicity of imidacloprid, triazophos, and deltamethrin on first-day fifth instar of *S*. *furcifera*.

Insecticide	Toxicity regression equation	LC_10_ (mg/L)	95% Confidence interval (95% CI)
Imidacloprid	*y* = 1.476*x*−0.845	0.506	0.313–0.976
Triazophos	*y* = 3.050*x*−4.526	11.581	0.362–19.943
Deltamethrin	*y* = 1.438*x*−2.601	8.276	5.055–11.49

### Transcriptome assembly and functional annotation

The cDNA libraries were constructed using RNA from all samples, with a mixture of *S*. *furcifera* bodies as the template. After filtering, 66.6 million clean reads were generated. These reads were assembled into 87,760 transcripts and 40,597 unigenes, with N50 lengths of 1,322, and 1741 nt, respectively (Table S2). In addition, we constructed 20 cDNA libraries from 20 samples using short sequencing, and performed an analysis of the differentially expressed genes (DEGs).

For functional annotation, all unigenes were searched in seven databases (Nr, Nt, Swiss-prot, KEGG, COG, Interpro, GO); 19,114 (47.08%) unigenes possessed homologous genes in the Nr protein database (Table S3). The species distribution of the best match for each sequence is shown in Fig. S1. The species distributions of the best matches for each sequence matched genes of the *Zootermopsis nevadensis* (17.38%) followed by *Halyomorpha halys* (10.65%), *Cimex lectularius* (10.35%), and *N*. *lugens* (3.18%) (Fig. S1). In addition, approximately 5.15, 20.0, and 37.10% of the unigenes were annotated in GO, COG, and KEGG based on sequence homologies, respectively (Table S3).

**Figure 1:**
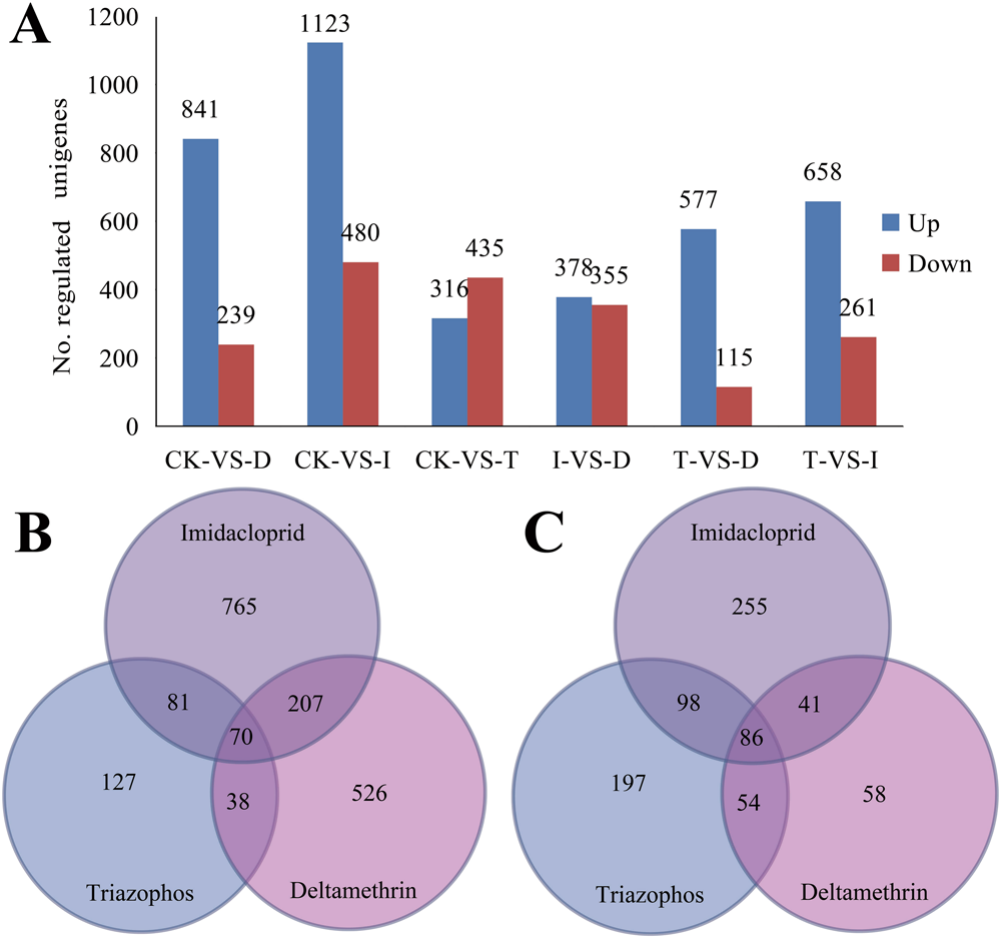
Differentially expressed genes (DEGs). (**A**) Histogram of the number of upregulated and downregulated unigenes among annotated unigenes in *Sogatella furcifera* in response to insecticide treatment. (**B**,**C**) Venn diagrams showing the number of genes commonly and differentially expressed in the deltamethrin, imidacloprid and triazophos treatment for upregulated (**B**) and downregulated (**C**) genes. CK, no insecticide treatment; D, deltamethrin treatment; I, imidacloprid treatment; and T, triazophos treatment.

In total, 1,080 unigenes were differentially regulated in response to deltamethrin treatment compared with the control (no insecticide treatment), including the upregulation of 841 unigenes and downregulation of 239 unigenes. Following imidacloprid treatment, 1,603 unigenes were differentially regulated, including the upregulation of 1,123 unigenes and downregulation of 480. Under triazophos treatment, 751 unigenes were differentially regulated, including 316 upregulated unigenes and 435 downregulated unigenes. In addition, compared with the triazophos treatment, deltamethrin and imidacloprid resulted in 692 and 919 unigenes being differentially regulated, respectively. Under imidacloprid treatment, 733 unigenes were differentially regulated compared with deltamethrin treatment (Fig. 1, Tables S4–S6). We GO-classified these differentially expressed genes (DEGs) to further understand their functions. The GO classifications were summarised in three main categories: biological process, cellular component, and molecular function, in response to treatment with the three insecticides (Figs S2–S4). In biological processes and molecular functions, “cellular processes and metabolic processes” and “catalytic activity” were both dominant in the response to treatment with the three insecticides. In cellular component, “cell and cell part,” “membrane,” and “membrane” were predominant following deltamethrin, imidacloprid, and triazophos treatment, respectively (Figs S2–S4).

Among these DEGs, 70 unigenes were commonly upregulated and 86 were commonly downregulated in response to the three insecticide treatments (Table S7; Fig. 1). Of these, only 24 upregulated unigenes had clear annotations (Table 2). Some genes were related to epidermal formation, such as laminin, larval cuticle protein, and fasciclin. Some genes may be involved in the detoxification, metabolism, and transport of these three insecticides, such as UDP-glucose:glycoprotein glucosyltransferase, and ABC transporter F family member 4-like. In addition, many genes were not annotated, and these may be also related to the development of resistance to these three insecticides in *S*. *furcifera*; further validation is needed in future studies.

**Table 2:** Upregulated differentially expressed genes (DEGs) with annotation in response to treatment with deltamethrin, imidacloprid, and triazophos.

GeneID	Blast nr
CL521.Contig1	Histone-lysine N-methyltransferase EZH2 [*Zootermopsis nevadensis*]
CL1986.Contig1	Sugar transporter 16 [*Nilaparvata lugens*]
CL2327.Contig4	Larval cuticle protein A3A-like [*Dufourea novaeangliae*]
Unigene21248	Trypsin-19 [*Nilaparvata lugens*]
Unigene22329	Protein scarlet-like [*Halyomorpha halys*]
Unigene28845	Fibrous sheath CABYR-binding protein-like [*Amyelois transitella*]
Unigene6833	Sialidase [*Toxocara canis*]
CL2624.Contig2	Asparagine–tRNA ligase, cytoplasmic [*Polistes dominula*]
Unigene4053	UDP-glucose:glycoprotein glucosyltransferase [*Tribolium castaneum*]
Unigene16047	Down syndrome cell adhesion molecule-like protein Dscam2 isoform X4 [*Cimex lectularius*]
CL112.Contig1	Aquaporin AQPAe.a [*Diaphorina citri*]
Unigene31078	Neurogenic locus Notch protein isoform X2 [*Halyomorpha halys*]
CL1521.Contig2	Extensin [*Diachasma alloeum*]
CL2605.Contig1	Lactoylglutathione lyase [*Culex quinquefasciatus*]
CL1747.Contig1	Lysosomal alpha-mannosidase [*Zootermopsis nevadensis*]
CL743.Contig2	Armadillo protein [*Gryllus bimaculatus*]
CL2549.Contig4	Mucin-5AC-like [*Halyomorpha halys*]
Unigene26956	Distal-less protein [*Nilaparvata lugens*]
CL1561.Contig2	Ribose-phosphate pyrophosphokinase 1 isoform X3 [*Halyomorpha halys*]
CL1975.Contig2	Splicing factor, arginine/serine-rich 2 [*Zootermopsis nevadensis*]
Unigene26928	Splicing factor, arginine/serine-rich 18 [*Zootermopsis nevadensis*]
Unigene7065	Chitinase [*Nilaparvata lugens*]
CL3475.Contig2	Myosin heavy chain, cardiac muscle isoform X2 [*Cimex lectularius*]
CL674.Contig2	Testican-1 [*Cimex lectularius*]
CL2426.Contig1	Centromere/kinetochore protein zw10-like protein [*Zootermopsis nevadensis*]
Unigene16316	Interference hedgehog-like isoform X5 [*Cimex lectularius*]
Unigene2429	Early nodulin-75-like, partial [*Tinamus guttatus*]
CL1210.Contig2	Fasciclin-3 [*Halyomorpha halys*]
Unigene17811	Laminin subunit alpha-1 [*Halyomorpha halys*]
Unigene6162	Laminin subunit alpha-1, partial [*Zootermopsis nevadensis*]
CL2907.Contig2	Zinc finger RNA-binding protein, partial [*Stegodyphus mimosarum*]
CL911.Contig1	Ras-related protein Rab-23 isoform X2 [*Halyomorpha halys*]
CL758.Contig1	Protein NDRG3 isoform X2 [*Halyomorpha halys*]
Unigene22467	Protein mab-21 [*Halyomorpha halys*]
CL2649.Contig2	ABC transporter F family member 4-like [*Diuraphis noxia*]
CL638.Contig3	Uncharacterised protein LOC107167888 [*Diuraphis noxia*]
Unigene1456	Protein white-like [*Halyomorpha halys*]
Unigene27433	Protein TANC2 isoform X7 [*Halyomorpha halys*]

### Identification of gene sequences encoding insecticide detoxification enzymes and insecticide targets

In order to identify the molecular mechanisms of deltamethrin, imidacloprid, and triazophos action in *S*. *furcifera*, sequences related to insecticide targets were assessed. As shown in Table 3, a number of sequences related to the insecticide targets, such as gamma-aminobutyric acid receptor, sodium channel and ryanodine receptor, acetylcholinesterase (AChE), nicotinic acetylcholine receptor (nAChR), chitinase, and chitin synthase were analysed. Of these, the sodium channel (VGSC) was a target of deltamethrin, nAChR was a target of imidacloprid, and AChE were targets of triazophos.

**Table 3:** Genes related to the insecticide targets.

Gene name	Number
Acetylcholinesterase	9
Gamma-aminobutyric acid receptor	5
Nicotinic acetylcholine receptor	10
Sodium channel	1
Ryanodine receptor	3
Chitinase	20
Chitin synthase	2

**Figure 2:**
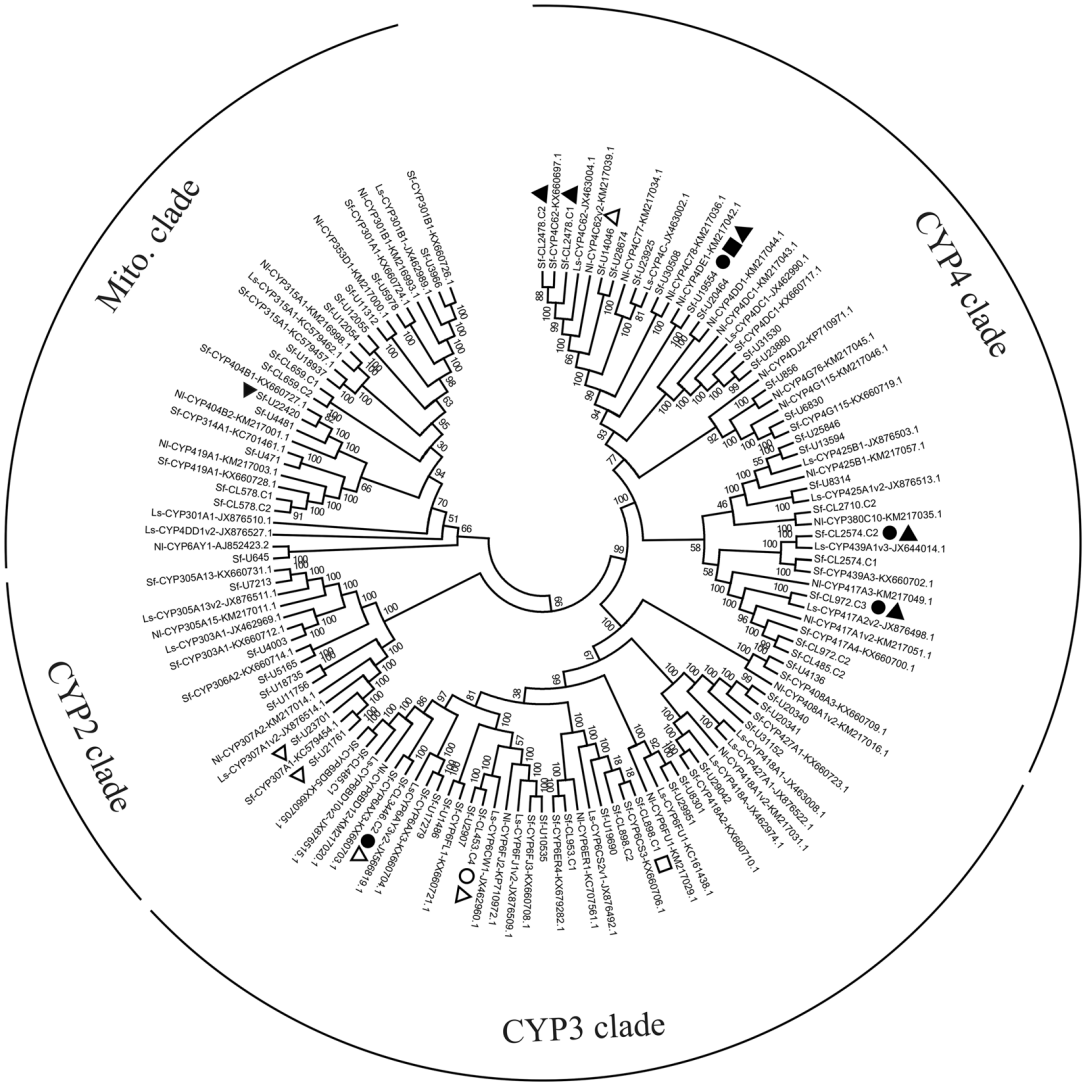
Phylogenetic analysis of putative P450 genes in *Sogatella furcifera* with those in other planthoppers. Ls, *Laodelphax striatellus*; Nl, *Nilaparvata lugens*; Sf, *S*. *furcifera*. Filled circles represent downregulation under triazophos treatment; unfilled circles present upregulation under triazophos treatment; filled triangles represent downregulation under imidacloprid treatment; unfilled triangles represent upregulation under imidacloprid treatment; filled squares represent downregulation under decamethrin treatment; and unfilled squares represent upregulation under decamethrin treatment.

Cytochrome P450 is an important metabolic enzyme involved in the synthesis and decomposition of exogenous and endogenous compounds^16,17^. It has dual roles in detoxification and activation following insecticide application^18,19^. These enzymes are divided in four major clades; CYP2, CYP3, CYP4, and mitochondrial (Mito)^20^. In this study, 59 unigenes were found to putatively encode P450 genes in *S*. *furcifera*. Based on the neighbour-joining tree used for phylogenetic analysis, these unigenes were assigned to CYP2 (7), CYP3 (18), CYP4 (21), and Mito (14) (Fig. 2). Compared with the control, one, one, and seven unigenes were upregulated under deltamethrin, triazophos, and imidacloprid treatment, respectively. Of these upregulated unigenes, two belonged to the CYP2 clade, four belonged to the CYP3 clade, and three belonged to the CYP4 clade (Fig. 2).

**Figure 3:**
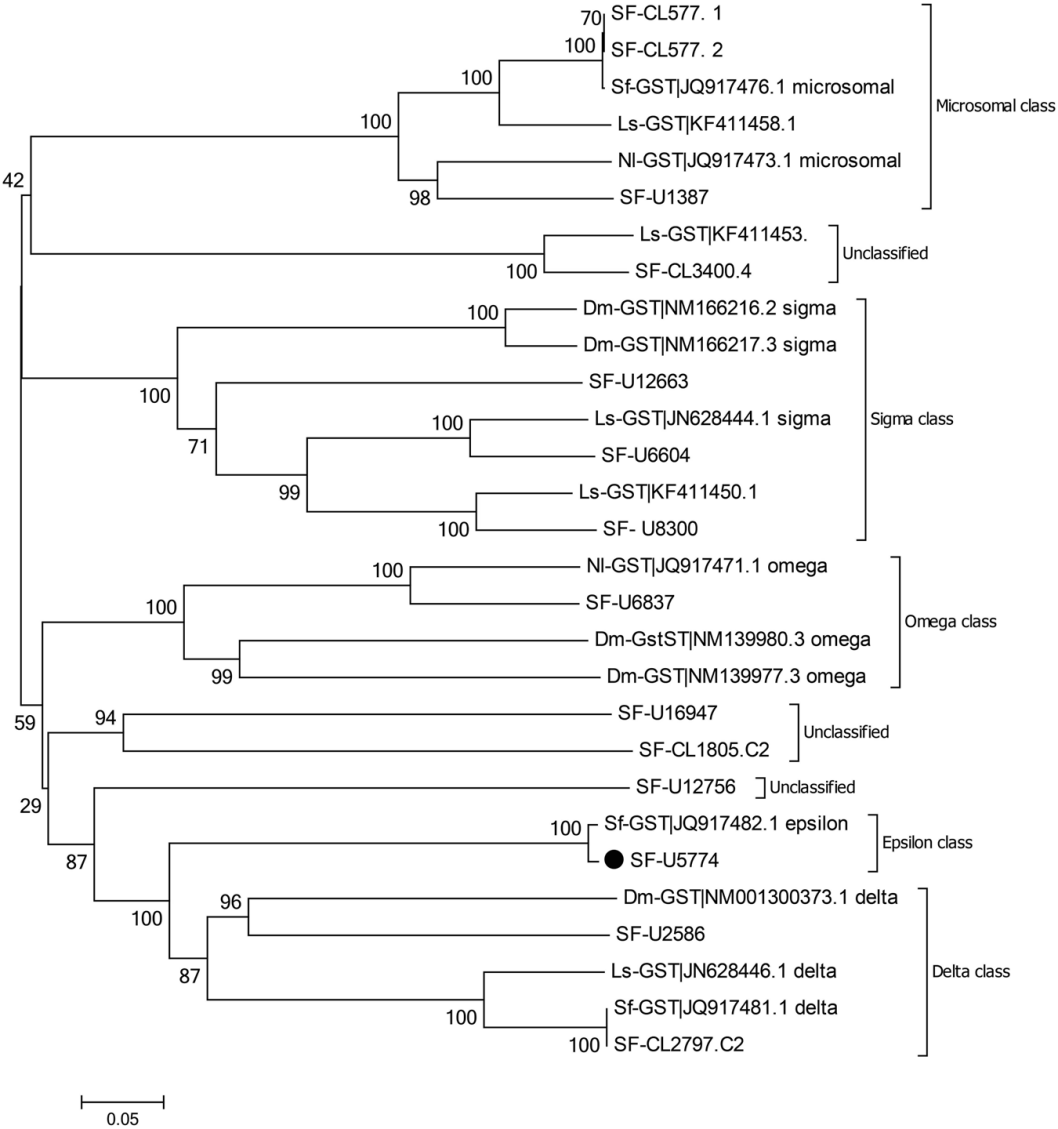
Phylogenetic analysis of putative glutathione S-transferase (GST) genes in *Sogatella furcifera* and those in other planthoppers. Ls, *Laodelphax striatellus*; Nl, *Nilaparvata lugens*; Sf, *S*. *furcifera*; Dm, *Drosophila melanogaster*. Circle represents upregulation under imidacloprid treatment.

GSTs belong to a super family of detoxifying enzymes, and thus have important roles in detoxification, protecting insects from various chemical insecticides. The GSTs can be divided into seven major classes in insects: sigma, zeta, omega, theta, microsomal, delta, and epsilon^21^. In this study, 24 unigenes were found to putatively encode GST genes in *S*. *furcifera* and these unigenes were assigned to the sigma (3), omega (1), microsomal (3), delta (2), and epsilon (1) classes (Fig. 3). However, only one unigene exhibited a two-fold difference in the expression of GST under imidacloprid treatment (Fig. 3).

Members of the multifunctional carboxylesterase (COE, EC 3.1.1.1) superfamily play important roles in xenobiotic detoxification, pheromone degradation, neurogenesis, and development regulation^22^, and can be subdivided into eight main subfamilies; including the α-esterases (ae), juvenile hormone esterases (JHE), β-esterases (be), gliotactins (gli), acetylcholinesterases (ace; AChE), neurotactin (nrt), neuroligins (nlg), and glutactin (glt) classes^23^. In this study, 60 unigenes were found to encode COE. Only one unigene, was putatively identified as carboxylesterase; this was upregulated under imidacloprid treatment compared with the control. In addition, compared with deltamethrin treatment, three putative carboxylesterase unigenes were upregulated and one unigene was downregulated under imidacloprid treatment, and two unigenes, designated as putatively designated juvenile hormone esterases, were upregulated. Under triazophos treatment, two unigenes designated as putatively designated carboxylesterase were downregulated compared to the deltamethrin treatment (Table 4).

**Table 4:** Summary of regulated carboxylesterase (COE) in *Sogatella furcifera* under different insecticide treatments.

Unigene ID	Log_2_ ratio	Versus	Annotation
U28681	1.05	CK vs I	Carboxylesterase-6 [*Laodelphax striatella*]
	−1.33	D vs I	
U14735	1.79	D vs I	Carboxylesterase-6 [*Laodelphax striatella*]
U18154	1.02	D vs I	Carboxylesterase-6 [*Laodelphax striatella*]
U14524	−2.46	D vs I	Carboxylesterase [*Laodelphax striatella*]
U23268	−2.02	T vs I	Carboxylesterase [*Laodelphax striatella*]
U25081	−1.76	T vs I	Carboxylesterase [*Laodelphax striatella*]
U10859	1.24	D vs I	Juvenile hormone esterase [*Nilaparvata lugens*]
U30294	1.52	D vs I	Juvenile hormone esterase [*Nilaparvata lugens*]

### Identification of gene sequences encoding ATP-binding cassette (ABC) transporters

ABC transporters comprise a large transporter family, and transport inorganic ions, sugars, amino acids, lipids, lipopolysaccharides, peptides, metals, xenobiotics, and chemotherapeutic drugs^13^. This family can be subdivided into eight major subfamilies (A–H) in insects^24^. In recent years, the expression of ABC transporters has been directly related to the generation of insect resistance^25,26^. In this study, 89 unigenes were found to encode ABC transporters. Compared with the control, one subfamily D unigene and one subfamily F unigene were upregulated under deltamethrin treatment, one subfamily F unigene was upregulated under imidacloprid treatment, and one subfamily G unigene was downregulated and one subfamily F unigene was upregulated under triazophos treatment. In addition, compared with triazophos treatment, one subfamily D unigene and two subfamily G unigenes were upregulated under imidacloprid treatment, and only one subfamily G unigene was upregulated under deltamethrin treatment (Table 5).

**Table 5:** Summary of regulated ATP-binding cassette (ABC) transporters in response to different insecticide treatments in *Sogatella furcifera*.

Unigene ID	Subfamily	Log_2_ fold-change	Versus
CL2649.2	ABCF	1.35	CK vs D
		1.57	CK vs I
		1.48	CK vs T
CL2980.2	ABCG	−1.48	CK vs T
		1.28	T vs I
U13482	ABCG	2.05	T vs I
U20841	ABCD	1.46	T vs I
		1.02	CK vs D
U30224	ABCG	1.12	T vs D

### Identification of gene sequences encoding heat shock proteins

HSPs are synthesised by organisms following exposure to physical, chemical, biological, and mental stimuli. They are widely present in all living organisms and play important roles in various physiological and biological processes^27^. HSPs are subdivided into six subfamilies based on their molecular weight and homology, including Hsp100, Hsp90, Hsp70, Hsp60, Hsp40, and small Hsps (sHsp)^28^. In this study, the expression of six unigenes from the Hsp70 subfamily and one unigene from the Hsp90 subfamily was upregulated, and one unigene from the sHsp subfamily was downregulated in response to imidacloprid treatment compared with the control, while only one unigene from the Hsp90 subfamily was upregulated more than two-fold in response to deltamethrin treatment. No changes in expression exceeding two-fold were observed for the Hsp genes with triazophos treatment (Table 6).

**Table 6:** Regulation of heat shock proteins (HSPs) under different insecticide treatments in *Sogatella furcifera*.

Subfamily	Unigene ID	Log_2_ fold change	Versus
Hsp90	CL2709.2	1.15	CK vs D
		1.71	CK vs I
Hsp70	U4279	3.66	CK vs I
	U10564	1.38	CK vs I
	CL3045.2	8.01	CK vs I
	CL3045.2	3.88	CK vs I
	CL2644.3	2.44	CK vs I
	CL2644.2	3.83	CK vs I
sHsp	CL2253.2	−1.63	CK vs I

### Validation of five common differentially expressed genes

To identify the common genes upregulated in response to treatment with the three insecticides (triazophos, imidacloprid, and deltamethrin), and to verify whether these genes show similar patterns of regulation in response to treatment with other insecticides (thiamethoxam, chlorpyrifos, pymetrozine, and abamectin), qPCR was performed. The candidate genes had significant sequence homologies to *N*. *lugens* trypsin-19, chitinase-9 and chitinase-10, *Diaphorina citri* aquaporin, and *Z*. *nevadensis* laminin subunit alpha-1.

**Figure 4:**
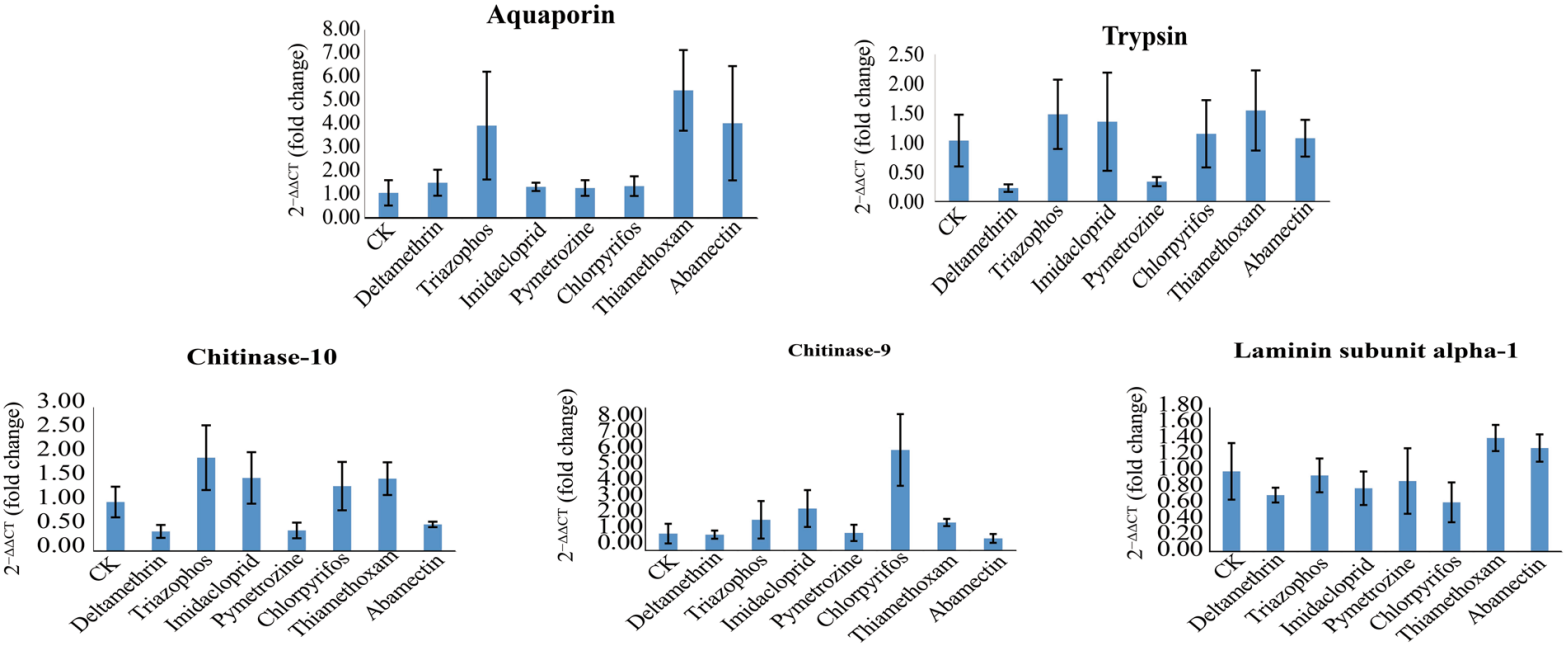
qPCR analysis of candidate common upregulated transcripts of *Sogatella furcifera* following insecticide treatment. The mean value ± SD was used to analyse the relative transcript levels under different treatments using the ΔΔCt method, with non-insecticide treatment (CK) as a reference.

The expression of *S*. *furcifera* trypsin was upregulated under triazophos, imidacloprid, chlorpyrifos, and abamectin stress, but was downregulated under deltamethrin and pymetrozine stress (Fig. 4). Aquaporin expression was only upregulated under triazophos, thiamethoxam, and abamectin stress (Fig. 4). Chitinase-10 and chitinase-9 transcript levels were upregulated under triazophos, imidacloprid, chlorpyrifos, and thiamethoxam stress, while upregulation of chitinase-9 expression was most obvious (six-fold increase) (Fig. 4). In addition, expression of the laminin subunit alpha-1 did not significantly change under insecticide stress, while only a small change in expression was observed under thiamethoxam and abamectin stress (Fig. 4).

Real-time qPCR analysis showed that trypsin, aquaporin, chitinase-10, and chitinase-9 were upregulated under triazophos and imidacloprid stress, with only a slight decrease in the expression of laminin subunit alpha-1 observed. In addition, the transcript levels of trypsin and chitin-10 were greatly decreased after deltamethrin stress. These differences are likely because of the sensitivity of real-time qPCR, which is less sensitive than next-generation sequencing.

## Discussion

*S*. *furcifera* is one of the most important migratory rice pests rice in Asia, and can transmit the Southern rice black-streaked dwarf virus resulting in substantial yield losses^1^. Insecticide use has always been an important way to control *S*. *furcifera*. Widespread application of insecticides leads to resistance and to the resurgence of the planthopper. The mechanisms responsible for insecticide resistance include high enzymatic activity, thus metabolising the insecticide quickly^29^.

Overexpression of P450s is a common mechanism of insecticide resistance^16,30^. The treatment of resistant and sensitive populations of *Tribolium castaneum* with sublethal concentrations of phosphine treatment resulted in the overexpression of two P450 genes (CYP6A14 and CYP346B-1) in the resistant population^31^. A CYP6α23-like gene was also found to be overexpressed in the spinosad-resistant populations of *Bactrocera oleae*^32^. In this study, eight unigenes were overexpressed in three treatments. Manual comparison of the BLASTx and Nr protein databases revealed that these unigenes were highly homologous with *S*. *furcifera* CYP307A1 (U21761, U23701), CYP6AX3 (CL3446.Contig2), CYP6CS3 (CL898.Contig1), *N*. *lugens* CYP4DC1 (Unigene23880), CYP427A1 (U20341), *Laodelphax striatella* CYP4C61 (U14046), and CYP6CW1 (CL453.Conting4). When imidacloprid was applied to *S*. *furcifera* at the LD_85_ dose, nine P450 genes were upregulated (including CYP6AX3, CYP6CS3, and CYP6CW1) and three were downregulated (including CYP4C61); however, there was very little difference between the expression of CYP4DC1 and CYP427A1^33^. Similar results were found in the present study, whereby the expression of CYP6AX3 (imidacloprid treatment), CYP6CW1 (imidacloprid and triazophos treatment), and CYP6CS3 (deltamethrin treatment) were upregulated when exposed to sublethal concentrations at the LC_10_. The expression of CYP4DC1, CYP427A1, and CYP4C61 was also significantly upregulated (>two-fold increase) following exposure to the LC_10_ of imidacloprid. CYP6AX3 expression was upregulated under imidacloprid stress, but was downregulated under triazophos stress. This may be because the response mechanisms under different levels or types of insecticide stress differ in *S*. *furcifera*. No P450 genes were found to be overexpressed at both high and low concentrations of cycloxaprid^34^. CYP307A1, a Halloween gene, may also be involved in ecdysteroidogenesis in *S*. *furcifera*^35^. After the application of sublethal concentrations of imidacloprid, CYP307A1 expression was also significantly increased. The role of CYP307A1 in *S*. *furcifera* resistance remains unknown, and further studies are needed.

Seven GSTs were upregulated in both chlorpyrifos- and fipronil-resistant strains of *Plutella xylostella*^36^. The expression of nine GST genes was upregulated following exposure of *S*. *furcifera* to sublethal doses (LD_20_) of imidacloprid^37^. In the present study, the expression of only one GST gene was significantly upregulated under imidacloprid treatment. BLASTx and protein manual database comparison found these unigenes to be highly homologous with *S*. *furcifera* GSTe1 (Unigene5774). This indicated that GSTe1 plays an important role in the resistance of *S*. *furcifera* to imidacloprid^37^.

COEs are important detoxifying enzymes in insects, and play important roles in detoxification and metabolism in insects^38^. Overexpression of COE can enhance the resistance of *Myzus persicae* to insecticides^38^. Transcriptome analysis showed that two COE genes were overexpressed in the imidacloprid-resistant population of *B*. *tabaci*^39^. In the present study, only one COE gene was significantly overexpressed under imidacloprid stress (>two-fold increase). AChE mutation is one of the main mechanisms of resistance to organophosphate and carbamate insecticides^40^. The overexpression of AChE is related to insecticide resistance. Stressing *S*. *furcifera* with high concentrations of cycloxaprid significantly increased the expression of AChE^34^. Omethoate resistance in *Aphis gossypii* is caused by mutation and overexpression of AChE^41^. However, no overexpression of AChE was found in response to triazophos treatment in the present study. These results suggest that *S*. *furcifera* is responsive to low concentrations of organophosphate insecticides via mutations in AChE. Most insects carry two AChE genes (including *N*. *lugens*)^42,43^. However, only AChE1 gene was annotated in the present study. This may be because the level of AChE1 transcription is much higher than that of AChE2^43^.

ABC transporters may play important roles in the transport and/or resistance of insecticides^25,26^. More than 20% of the ABC transporters (total of 40 ABC transporters) were overexpressed in *L*. *striatellus* populations resistant to chlorpyrifos, deltamethrin, and imidacloprid^14^. One ABCA subfamily and four ABCG subfamily genes were significantly upregulated in imidacloprid-treated *B*. *tabaci*^44^. In the present study, five ABC transporters (one ABCD subfamily, three ABCF subfamily, and one ABCG subfamily) were significantly upregulated. However, there was a significant decrease in the transcriptional level of the ABCG subfamily with triazophos treatment (Table 5). This may be because this ABC transporter gene is not involved in the transmission or resistance of insecticides, but is involved in other biological processes in *S*. *furcifera*. At present, although the overexpression of ABC transporters has been observed in many resistant populations of insects, the mechanism of resistance is still unclear.

As molecular chaperones, Hsps may be induced by insecticides, and they also contribute to insecticide resistance^15^. Nazir *et al*.^45^ showed strong Hsp70 expression in the Captafol-exposed larvae of *D*. *melanogaster*. In addition, two Hsp70s were upregulated in a chlorpyrifos-resistant population of *Plutella xylostella*, whereas six sHsps were downregulated^46^. In the present study, six Hsp70s were upregulated with imidacloprid treatment, whereas one sHsp was downregulated. In the imidacloprid and deltamethrin treatment groups, one Hsp90 was upregulated, respectively. Studies have also shown that Hsps respond to different concentrations of insecticides in different ways. For example, the expression of one Hsp70 and one sHsp was downregulated in *S*. *furcifera* with low concentrations of cycloxaprid treatment, but was upregulated with high concentrations^34^. However, in this study, the response of *S*. *furcifera* was only assessed using low concentrations of insecticide and the differences between different insecticides were compared; however, the effects of different concentrations were not compared in this experiment. In future studies, we aim to study the response of *S*. *furcifera* to different concentrations of insecticides to clarify the response mechanism of *S*. *furcifera* under different levels of insecticide stress.

In this study, 40,597 unigenes with a mean length of 955 bp were assembled from the transcriptome analysis (Table 2). Among these, 1,080, 1,603, and 751 unigenes were regulated under deltamethrin, imidacloprid, and triazophos stress, respectively. In addition, many unigenes were regulated in response to the different insecticides. These DEGs may play different roles under stress induced by different insecticides. The results of the present study suggest that the mechanisms of insects respond to different insecticides stress differently, and the response to the same insecticide stress is also regulated by multiple genetic pathways. We were also interested in unigenes that were commonly upregulated or downregulated in response to three insecticides; 156 unigenes were regulated under deltamethrin, imidacloprid, and triazophos stress (Table S7). These common regulated unigenes may play similar roles in insect responses to different insecticide stresses. To test the hypothesis that these genes are expressed in the same way in response to stress from other insecticides, we screened five candidate genes from a common regulated gene for qPCR analysis with imidacloprid, thiamethoxam, triazophos, deltamethrin, pymetrozine, chlorpyrifos, and abamectin stress. However, the results were not consistent with our hypothesis, and these candidate genes were upregulated only in response to some insecticides (Fig. 4). In mosquitoes, trypsin is secreted by the midgut, and functions primarily in digestion and activating other zymogens^47^. The gene encoding trypsin was highly expressed in the deltamethrin-resistant population of *Culex pipiens pallens*^48^. In this study, trypsin expression was upregulated under triazophos, imidacloprid, chlorpyrifos, and abamectin stress, implying that trypsin may be related to resistance or to the response to stress induced by triazophos, imidacloprid, chlorpyrifos, and abamectin in *S*. *furcifera*. Aquaporins are important proteins that regulate the osmotic balance in insects^49^ and also play an important role in cold and desiccation resistance^50,51^. At present, there are no reports on the transcriptional level of aquaporin under insecticide stress. In this study, aquaporin expression was upregulated under triazophos, thiamethoxam, and abamectin stress. It is possible that increased aquaporin expression in *S*. *furcifera* is a response to insecticidal stress, which enhances water recycling, and reduces the damage at target sites. In the process of insect molting, chitinase degrades chitin in the old epidermis and peritrophic membrane into a soluble form that can be partially reabsorbed, and is used for the synthesis of new epidermis and peritrophic membrane^52^. Chitin-degrading enzymes include the chitinases, β-*N*-acetylglucosaminidases, and chitin deacetylase^52,53^. In this study, the transcript levels of chitinase-10 and chitinase-9 were upregulated under triazophos, imidacloprid, chlorpyrifos, and thiamethoxam stress. Detoxification and metabolism of insects using insecticides is a process that consumes energy. We hypothesised that the expression of chitinase would be increased, and that this could be explained by the degradation of the old epidermal or peritrophic membrane, providing energy for the metabolism of detoxifying enzymes. However, there is no evidence to support this hypothesis. Upregulation of chitin deacetylase was observed in *N*. *lugens* under triazophos stress^54^. Therefore, the mechanism of the insect response to insecticide stress needs further study. Among these common DEGs, some are associated with epidermal formation, such as laminin^55^, larval cuticle protein^56^, and fasciclin^57^. Overexpression of these genes may alter the epidermal permeability of *S*. *furcifera* and their response to insecticide stress. In addition, according to GO classification of DEGs, the membrane plays a dominant role in cell components, and also shows that epidermal permeability may play an important role in insecticide resistance. These common DEGs may also play important roles in insecticide cross resistance.

## Conclusions

In conclusion, some P450s, GSTs, COEs, Hsps, and ABC transporters were highly expressed at different levels under insecticide stress. These proteins may be involved in the metabolism and translocation of insecticides in *S*. *furcifera*. In addition, stress induced by the three insecticides resulted in a number of common DEGs. For example, laminin, fasciclin, larval cuticle protein, and other genes that affect epidermal formation. These genes may be involved in the altered epidermal permeability of *S*. *furcifera*, thus contributing to insecticide resistance.

## Materials and Methods

### Insects and insecticides

*S*. *furcifera* individuals were collected from a rice field in Huaxi, Guiyang, Guizhou, China, in 2013 and maintained in the laboratory, without any exposure to insecticides, on rice seedlings at 25 ± 1 °C and 70 ± 10% relative humidity with a 16:8 h (L: D) photoperiod. First-day fifth instar nymphs were used in the study. Imidacloprid (96.4%, technical formulation), chlorpyrifos (95.6%, technical formulation), and triazophos (83.56%, technical formulation) were obtained from Guangxi Tianyuan Biochemistry Co., Ltd (Guangxi, China). Deltamethrin (98%, technical formulation) was obtained from Shanghai superior Industrial Co., Ltd (Shanghai, China); thiamethoxam (96%, technical formulation) was obtained from PFchem Co., Ltd (Nanjing, China); abamectin (96.4%, technical formulation) was obtained from Shandong Qilu King-Phar Pharmaceutical Co., Ltd (Shandong, China); and pymetrozine (95.25%, technical formulation) was obtained from Yinbang Chemicals Co., Ltd (Shaoxing, China).

### Bioassay and sample collection

The rice cultivar Taichung Native 1 (TN-1), bred for tillering, was used in this study. A bioassay was performed according to modified version of the rice stem dipping method^58,59^. Insecticide was dissolved in acetone and then geometric diluted in distilled water to generate five concentrations. Briefly, rice stems, including the roots, were pulled from the soil and washed thoroughly. Fifteen-centimetre-long stems, as measured from the base, were cut and air-dried to remove any excess water. Two rice seedlings were immersed in each concentration of each insecticide for 30 s; rice stems treated with distilled water were used as a control. The rice stems were then placed in the shade to dry, whilst moistened cotton was used to wrap the roots. The rice stems were then placed into glass tubes (300 mm height × 30 mm diameter) that were open at both ends. Then, 20 fifth-instar first-day nymphs were introduced into each glass tube using a suction device, and five repeats were performed for each concentration. The treated insects were maintained at 25 ± 1 °C and 70 ± 10% relative humidity with a 16:8 h (L: D) photoperiod in an artificial climate box, and mortality counts were recorded after 72 h. Bioassay data analyses were performed using SPSS, version 17.0 (SPSS, Chicago, IL, USA), and the 10% lethal concentration (LC_10_) was calculated. The LC_10_ of each insecticide (imidacloprid, triazophos, and deltamethrin) and distilled water as a control were applied to the *S*. *furcifera* using the fifth-instar first-day nymphs. After 48 h, 15 surviving insects were randomly collected for subsequent RNA isolation, transcriptome sequencing, with five replications per treatment. In addition, the fifth-instar first-day nymphs were treated with imidacloprid, triazophos, deltamethrin, thiamethoxam, pymetrozine, chlorpyrifos, and abamectin, using the same method. After 48 h, 15 surviving insects were randomly collected for qRT-PCR. The LC_10_ values of thiamethoxam, pymetrozine, chlorpyrifos, and abamectin against *S*. *furcifera* were 0.33, 1.9621, 1.5295, and 1.0446 mg/L, respectively, as previously described^60^.

### cDNA library construction and Illumina sequencing

Total RNA was extracted from 15 insects using TRIzol Reagent (Invitrogen, Life Technologies, Carlsbad, CA, USA) following the manufacturer’s instructions. RNA was then treated with DNase I (Invitrogen, Life Technologies, Carlsbad, CA, USA). Oligo (dT) beads were used to isolate poly(A) + mRNA, which was fragmented to 150 bp. First-strand cDNA was synthesised using short fragments of random hexamer primers as templates and second-strand cDNA was synthesised using DNA polymerase I and RNase H. Short fragments were purified with the QiaQuick PCR extraction kit and eluted with EB buffer for end-repair and the addition of poly(A). Suitable fragments were selected, purified, and subsequently PCR-amplified to create the final cDNA library template. The quality of the final cDNA library was assessed with the Agilent 2100 Bioanalyzer system (Agilent, USA), which was sequenced by BGI (Shenzhen, China) using Illumina HiSeq2000 (Illumina, San Diego, CA, USA).

### *De novo* assembly and annotation

Before assembly, we removed the low-quality and adaptor sequences from the raw data. These clean reads were then *de novo* assembled into unigenes with Trinity software^61^. Subsequently, unigenes >200 bp were annotated using BLASTx with the following protein databases; non-redundant protein database (Nr), nucleotide sequence databases (Nt), Swiss-Prot, Kyoto Encyclopedia of Genes and Genomes (KEGG), and Cluster of Orthologous Groups (COG) (e-value < 10^−5^), to identify proteins with high sequence similarity and to assign putative functional annotations. Next, we obtained Gene Ontology (GO) annotations of the unigenes using the Blast2GO program^62^.

### Differentially expressed genes and phylogenetic analysis

Based on the transcriptomic data, we quantified the expression levels of genes using the BGIseq-500 platform (BGI, Wuhan, China, http://www.seq500.com/en/) with five biological replicates of each treatment. Unigene transcripts were quantified with the RSEM tool^63^. Quantitative results are expressed in fragments per kilobase of transcript per million mapped reads (FPKM)^64^, and the formula was as follows: FPKM (A) = (10,00,000 × C × 1,000)/(N × L), where FPKM (A) is the transcript quantification of gene A, C is the number of reads uniquely aligned to gene A, N is the total number of reads uniquely aligned to all genes, and L is the number of bases in gene A. The FPKM method eliminates the influence of gene lengths and sequencing discrepancies when calculating gene expression. The NOISeq^65^ method was used to screen differentially expressed genes (DEGs) between two groups using the following criteria as default: fold-change ≥ 2.0 (Log_2_ ratio ≥ 1.0) and diverge probability ≥ 0.7. Phylogenetic trees were constructed with MEGA v.6, to predict classification of the unigenes encoding P450s and GSTs. These were constructed with the neighbour-joining (NJ) method, using the bootstrap test with 1,000 replicates^66^.

### Identification of genes

We identified sequences associated with insecticide detoxification, stress responses, and insecticide targets from annotated transcripts by conducting searches against the Nr annotation databases with a cut-off E-value < 10^−5^. The complete coding region was confirmed by the ORF finder (http://www.ncbi.nlm.nih.gov/gorf/gorf.html) and protein BLAST results.

### qRT-PCR validation

Five sequences from the DEGS that were commonly upregulated in response to the three insecticides were selected to examine the variation in their expressions following the application of seven insecticides, namely neonicotinoids (imidacloprid and thiamethoxam), organophosphorus (triazophos and chlorpyrifos), pyrethroids (deltamethrin), macrolides (abamectin), and pyridine azomethine derivatives (pymetrozine), at their LC_10_ values. Total RNA was extracted using TRIzol reagent (Invitrogen, Life Technologies, Carlsbad, CA, USA). The RNA concentration was adjusted with diethyl pyrocarbonate (DEPC)-treated H_2_O to 0.8 µg/µl, and 0.8 µg of RNA was reverse transcribed in a 20 µl reaction system using the PrimeScript™ RT reagent Kit with gDNA Eraser (TaKaRa, Japan), with 18S rRNA (GenBank accession number: HM017250) as an internal control. Specific primer pairs for each gene were designed using Primer Premier 6 (Table S1). Real-time quantitative PCR (qPCR) was performed using a CFX96™ real-time quantitative PCR system (BioRad, Hercules, CA, USA) with the iTaq™ Universal SYBR® Green Supermix (Bio-Rad, Hercules, CA, USA), according to the manufacturer’s protocol. Each qPCR was conducted in a 20 μl mixture containing 1 μl of sample cDNA, 1 μl of each primer (10 μM), 7 μl of DEPC H_2_O, and 10 μl of 2 ×iTaq™ Universal SYBR® Green Supermix. The qPCR cycling parameters were as follows: 95 °C for 2 min, followed by 40 cycles of 95 °C for 30 s and 60 °C for 30 s, and melting curve generation was performed from 65 to 95 °C. To check reproducibility, the qPCR for each sample was performed in three technical replicates and three biological replicates. The comparative 2^−ΔΔCT^ method^67^ was used to calculate the relative quantification. Data analyses were performed using SPSS version 17.0 (SPSS, Chicago, IL, USA). The relative expression of each target gene was calculated with one-way nested analysis of variance (ANOVA) and Duncan’s new multiple range tests (*P* < 0.05).

### Availability of data and materials

The data generated and transcripts obtained were deposited at NCBI as the SRA accession SRP116252 and TSA accession GFWP00000000.1. The data and material are also provided as supplementary data.

## Electronic supplementary material

Supplementary Figure S1-4

Supplementary Tables S1-3

Supplementary Table S4

Supplementary Table S5

Supplementary Table S6

Supplementary Table S7
