# Supplementary material for: Comparative transcriptome analysis of Sogatella furcifera (Horváth) exposed to different insecticides
Source: Sci Rep. 2018 Jun 8;8:8773. doi: 10.1038/s41598-018-27062-4 (PMC5993722; doi:10.1038/s41598-018-27062-4)
Supplement: Supplementary file 1 — Supplementary Figure S1-4 [file 41598_2018_27062_MOESM1_ESM.pdf]

# Comparative transcriptome analysis of *Sogatella furcifera* (Horváth) exposed to different insecticides

**Cao Zhou[1], Hong Yang[1, 2*], Zhao Wang[1, 3], Gui-yun Long[1], and Dao-chao Jin[1]**

[1]Institute of Entomology, Guizhou University; Provincial Key Laboratory for Agricultural Pest Management of Mountainous Regions, Guiyang 550025, People's Republic of China

[2]College of Tobacco Science of Guizhou University, Guiyang, 550025, People's Republic of China

[3]College of Environment and Life Sciences, Kaili University, Kaili, 556011, People's Republic of China

[*] Corresponding author E-mail address: axyridis@163.com.

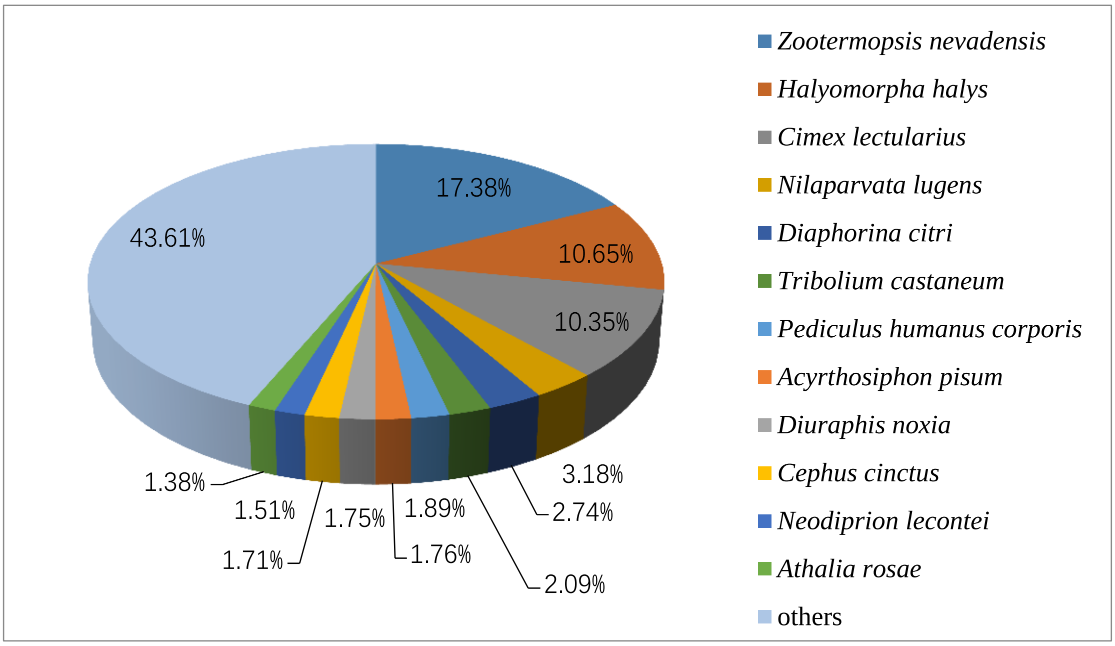


Figure S1. **Species distribution of the BLASTX results of *S. furcifera***. This figure shows the species distribution of unigene BLASTX results against the nr protein database with a cutoff E-value<$10^{-5}$ and the proportions of each species. Different colors represent different species. Species with proportions of more than 1% are shown.

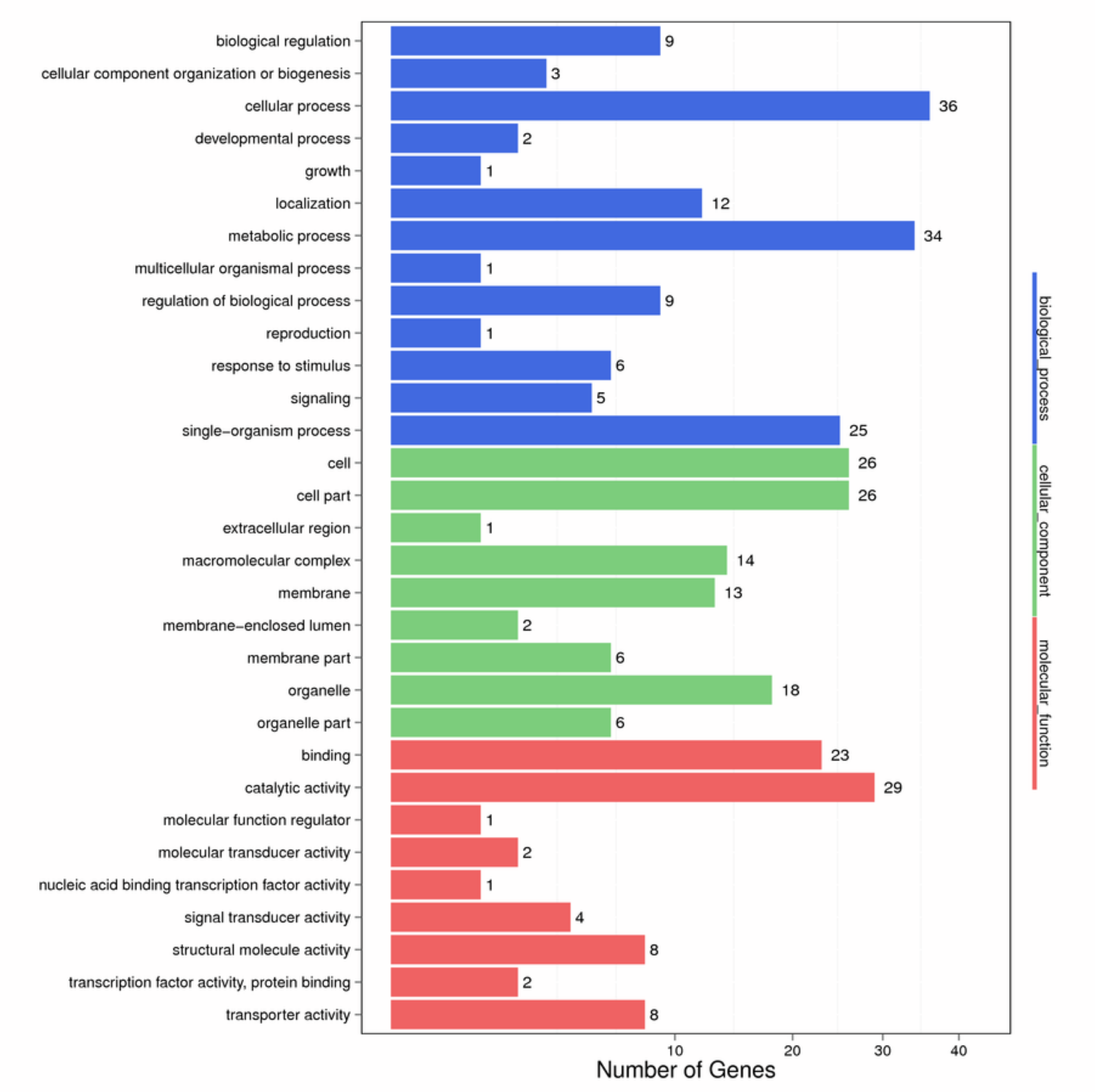


Figur S2 Gene ontology classification of differentially expressed genes with deltamethrin treatment. The genes are summarized in three main categories: biological process, cellular component and molecular function. The X-axis represent the number of genes in that main category.

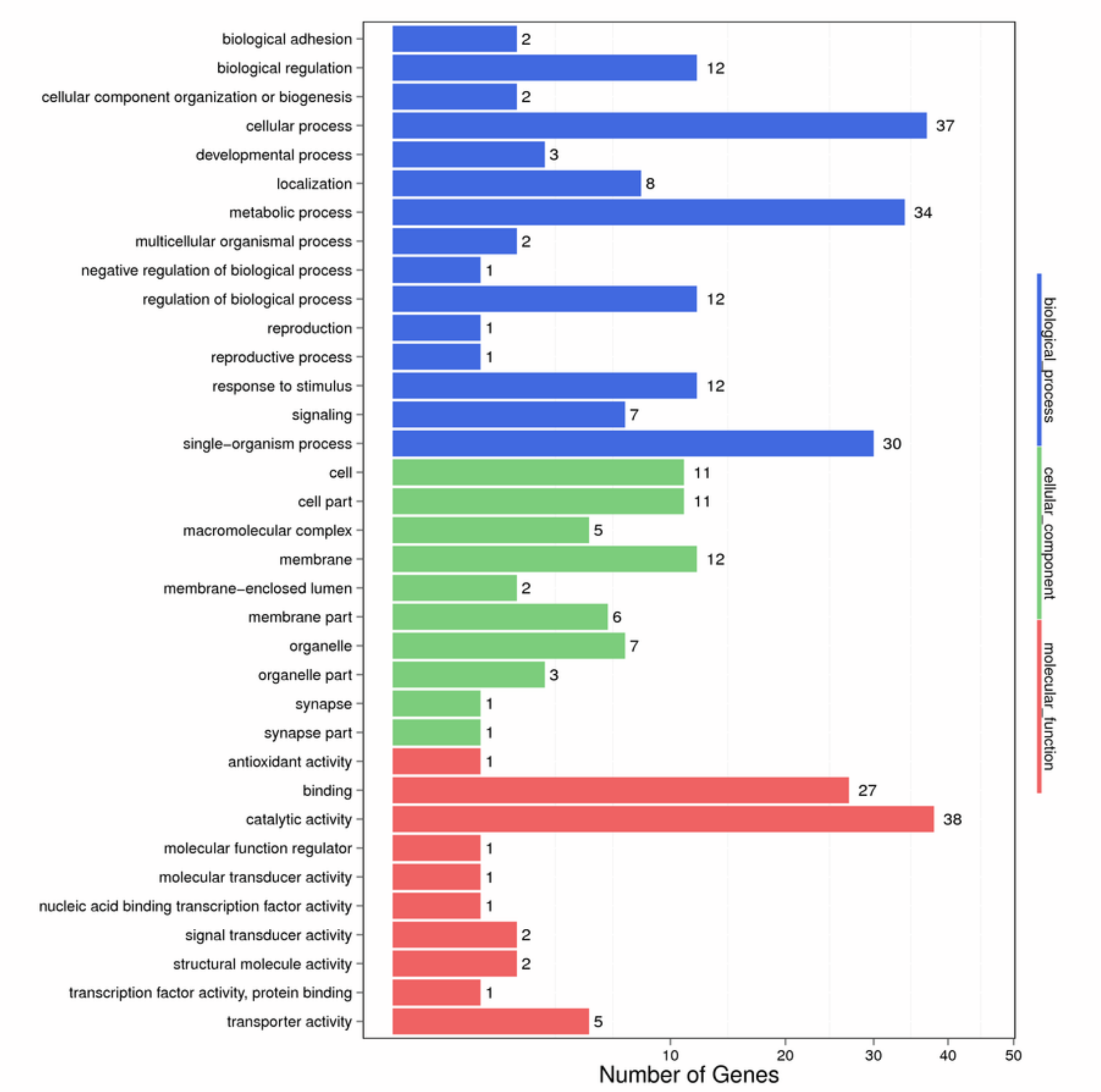


Figur S3 Gene ontology classification of differentially expressed genes with imidacloprid treatment. The genes are summarized in three main categories: biological process, cellular component and molecular function. The X-axis represent the number of genes in that main category.

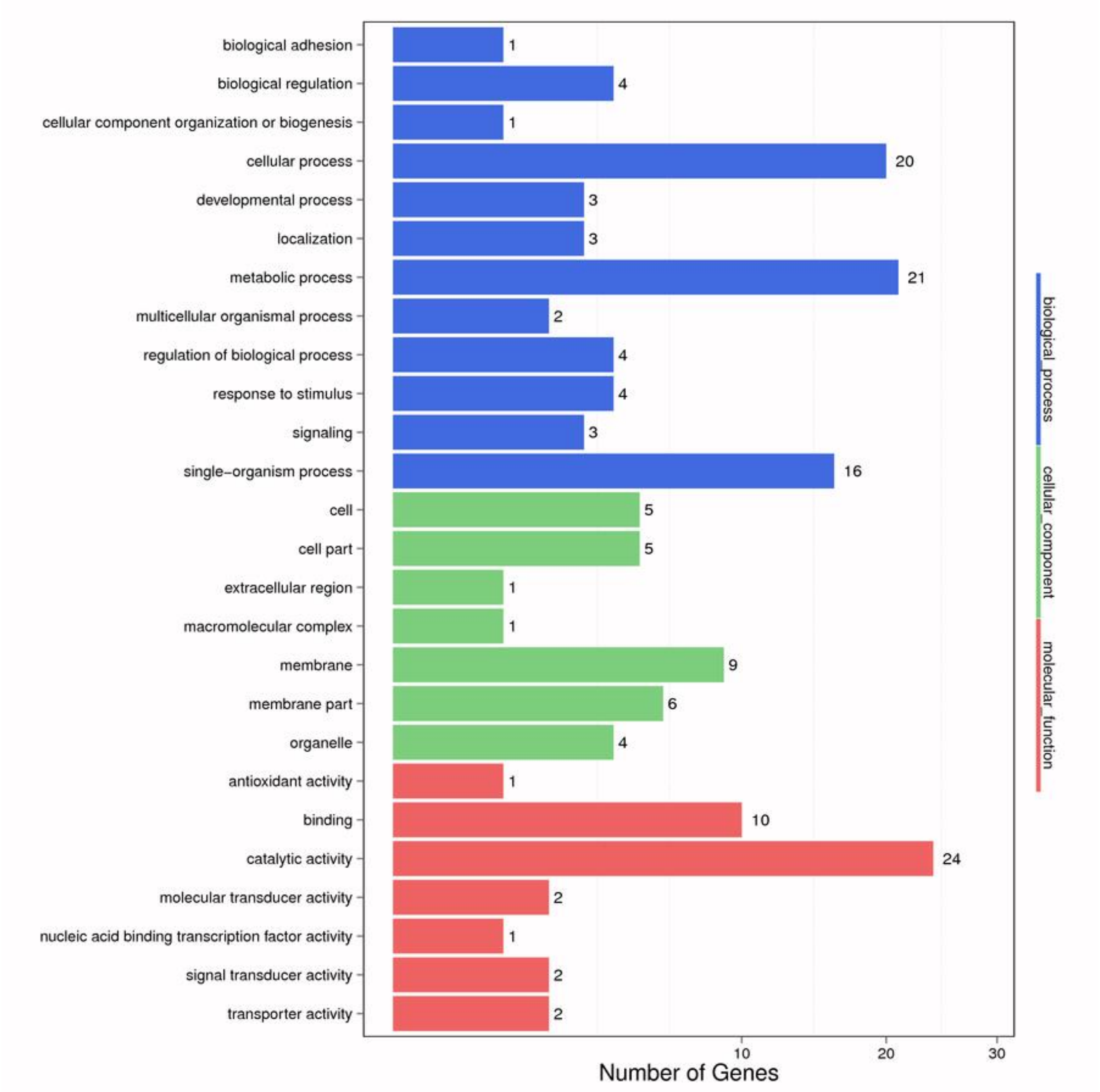


Figur S4 Gene ontology classification of differentially expressed genes with triazophos treatment. The genes are summarized in three main categories: biological process, cellular component and molecular function. The X-axis represent the number of genes in that main category.
