# Supplementary material for: Comparative transcriptome analysis of Sogatella furcifera (Horváth) exposed to different insecticides
Source: Sci Rep. 2018 Jun 8;8:8773. doi: 10.1038/s41598-018-27062-4 (PMC5993722; doi:10.1038/s41598-018-27062-4)
Supplement: Supplementary file 2 — Supplementary Tables S1-3 [file 41598_2018_27062_MOESM2_ESM.pdf]

Comparative transcriptome analysis of *Sogatella furcifera* (Horváth) exposed to different insecticides

Cao Zhou[1], Hong Yang[1, 2*], Zhao Wang[1, 3], Gui-yun Long[1], and Dao-chao Jin[1]

[1]Institute of Entomology, Guizhou University; Provincial Key Laboratory for Agricultural Pest Management of Mountainous Regions, Guiyang 550025, People's Republic of China

[2]College of Tobacco Science of Guizhou University, Guiyang, 550025, People's Republic of China

[3]College of Environment and Life Sciences, Kaili University, Kaili, 556011, People's Republic of China

[*] Corresponding author E-mail address: axyridis@163.com.

Table S1 The specific primers in qRT-PCR

Unigene ID	Annotation	Primer (5'-3')
Unigene 21248	trypsin-19 (*Nilaparvata lugens*)	F: AAGCGGCAAGCAATGGAAGT
		R: GCAGCATACACCGAACATCTGT
CL112. Contig1	aquaporin 2-like protein (*Bactericera cockerelli*)	F: GTCGTCATCGCACTCTCCTTC
		R: GCCGCAGATCCACCAATAGC
Unigene 7065	chitinase-10 (*Nilaparvata lugens*)	F: TACGACCGAGTAGTTGCCATGA
		R: AGTTCACCTGCCAGCACTTG
	chitinase-9 (*Nilaparvata lugens*)	F: CGTTCGCCGATGTCGTTCA
		R: GCCACACTGAGCAACACCTT
Unigene 6162	laminin subunit alpha-1 (*Pogonomyrmex barbatus*)	F: CTGGCAAACGGTGTGAAAGATG
		R: GTGATTCCTGGCCGACAGTTG

Table S2. Summary of *S. furcifera* transcriptome assembly

	Total Number	Total Length	Mean Length	N50	N70	N90	GC(%)
transcripts	87, 760	59, 686, 631	680	1, 322	600	247	36.88
unigene	40, 597	38, 797, 336	955	1, 741	971	357	37.84

Table S3. Functional annotation result

**Values**	**Total**	**Nr**	**Nt**	**Swiss-prot**	**KEGG**	**COG**	**Interpro**	**GO**	**Overall** [a]
Number	40,597	19,114	10,199	15,783	15,060	8,120	14,770	2,091	21,556
Percentage	100%	47.08%	25.12%	38.88%	37.10%	20.00%	36.38%	5.15%	53.10%

a: The total number of Unigene on any database annotation in seven databases.
